# Brain Stem Ischemic Stroke Associated with Anaphylaxis

**DOI:** 10.7759/cureus.2289

**Published:** 2018-03-08

**Authors:** Luis A Robles, Antonio F Matilla

**Affiliations:** 1 Section of Neurosurgery, Hospital Cmq; 2 Section of Internal Medicine, Hospital Cmq

**Keywords:** lateral medullary syndrome, wallenberg syndrome, ischemic stroke, anaphylaxis

## Abstract

Anaphylaxis is a serious allergic reaction that may have different manifestations including hypotension. It is reported that vertebral artery hypoplasia (VAH) may be present in up to 20% of the general population. Previous studies have demonstrated that patients with VAH have a higher risk of developing an ischemic stroke in the area supplied by this hypoplastic artery. This paper describes the case of a patient with preexistent VAH who presented with lateral medullary syndrome associated with a hypotensive episode secondary to anaphylaxis. To the best of the authors' knowledge, this association has not previously reported.

## Introduction

Anaphylaxis is a serious, systemic, and life-threatening allergic reaction which most commonly is caused by foods, insect stings, and medications. Anaphylaxis is rapid in onset and it is characterized by respiratory and circulatory dysfunction, and usually associated with cutaneous and mucosal changes [1]. Anaphylaxis may be lethal when the circulatory and respiratory systems are severely compromised. In these patients, when death occurs, it is usually the result of shock.

Congenital variations in the size of the vertebral arteries are frequently observed in the normal population; this variation may range from asymmetry to severe hypoplasia of one vertebral artery. Previous reports suggested that HVA is considered when the diameter of the vertebral artery is less than 2-3 mm. A study by Park et al. showed a high incidence of VAH in patients with posterior circulation strokes [2]. In this study, 51% of patients with vertebral artery territory stroke showed ipsilateral VAH, which was too high compared to the control group, in which VAH was observed in 26% of cases. In another paper, Thierfelder et al. studied the perfusion in the PICA territory in cases of VAH. This study showed that VAH can lead to hypoperfusion in the area supplied by this artery [3]. More studies have demonstrated the same findings [4-5].

This paper presents the case of a patient who experienced a lateral medullary syndrome caused by an ischemic stroke which was associated with anaphylaxis. To the best of the authors' knowledge, cases like this have not been previously described.

## Case presentation

This is a 55-years-old male, with no significant past medical history. His condition started after eating a salad in a restaurant. He mentioned that he had eaten the same salad several months before and experienced a mild allergic reaction, he thought that the ingredient causing the allergy was the dressing. Several minutes after eating this food, he felt several symptoms suggestive of an allergic reaction including itchy and watery eyes, sneezing, runny nose, cough and trouble breathing. On admission, he was reported hypotensive with a blood pressure of 80/40 mmHg. He was started on intravenous fluids, steroids, and antihistaminics. One hour later; he experienced dizziness, vomiting, diplopia, difficulty in swallowing, and tingling on the right limbs.

**Figure 1:**
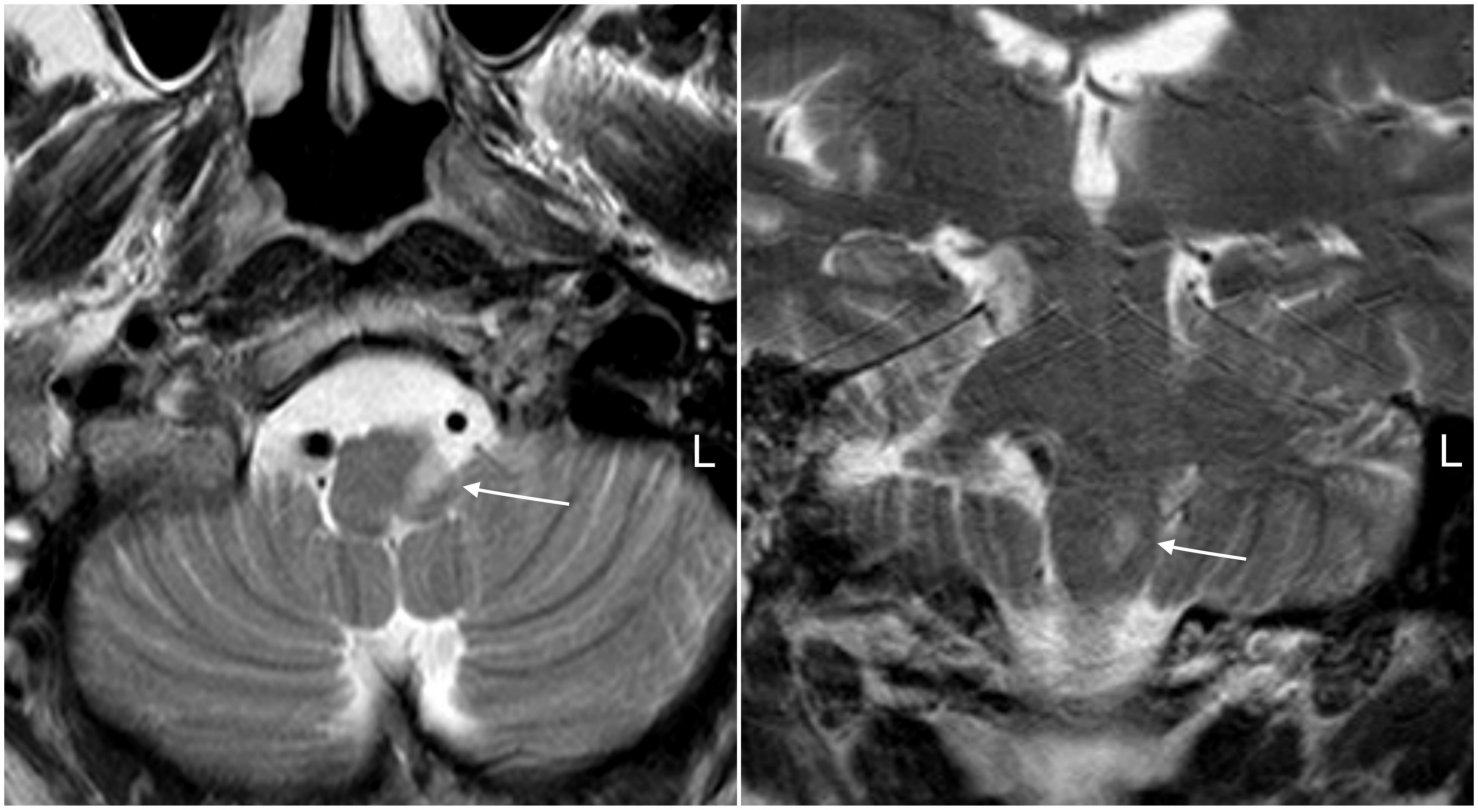
Brain magnetic resonance imaging (MRI) Left: T2 sequence axial MRI displays a hyperintense area located in the left lateral medullary area (arrow). Note the diameter difference between both the vertebral arteries. Right: T2 sequence coronal showing the same finding.

On initial examination, he was alert, oriented, and presented with slight dysarthria. The patient’s bilateral eye movements were normal. The patient presented with left Horner syndrome which included partial ptosis and miosis. In addition, the patient demonstrated left paresis of the palate. Motor function was normal in upper and lower extremities. Myotatic reflexes were normal. The patient demonstrated decreased sensation to pain and temperature to the right upper and lower extremities. An initial computed tomography (CT) scan was performed which was normal. The next day, a magnetic resonance imaging (MRI) was ordered that showed a left lateral medullary ischemic stroke (Figure 1). 

**Figure 2:**
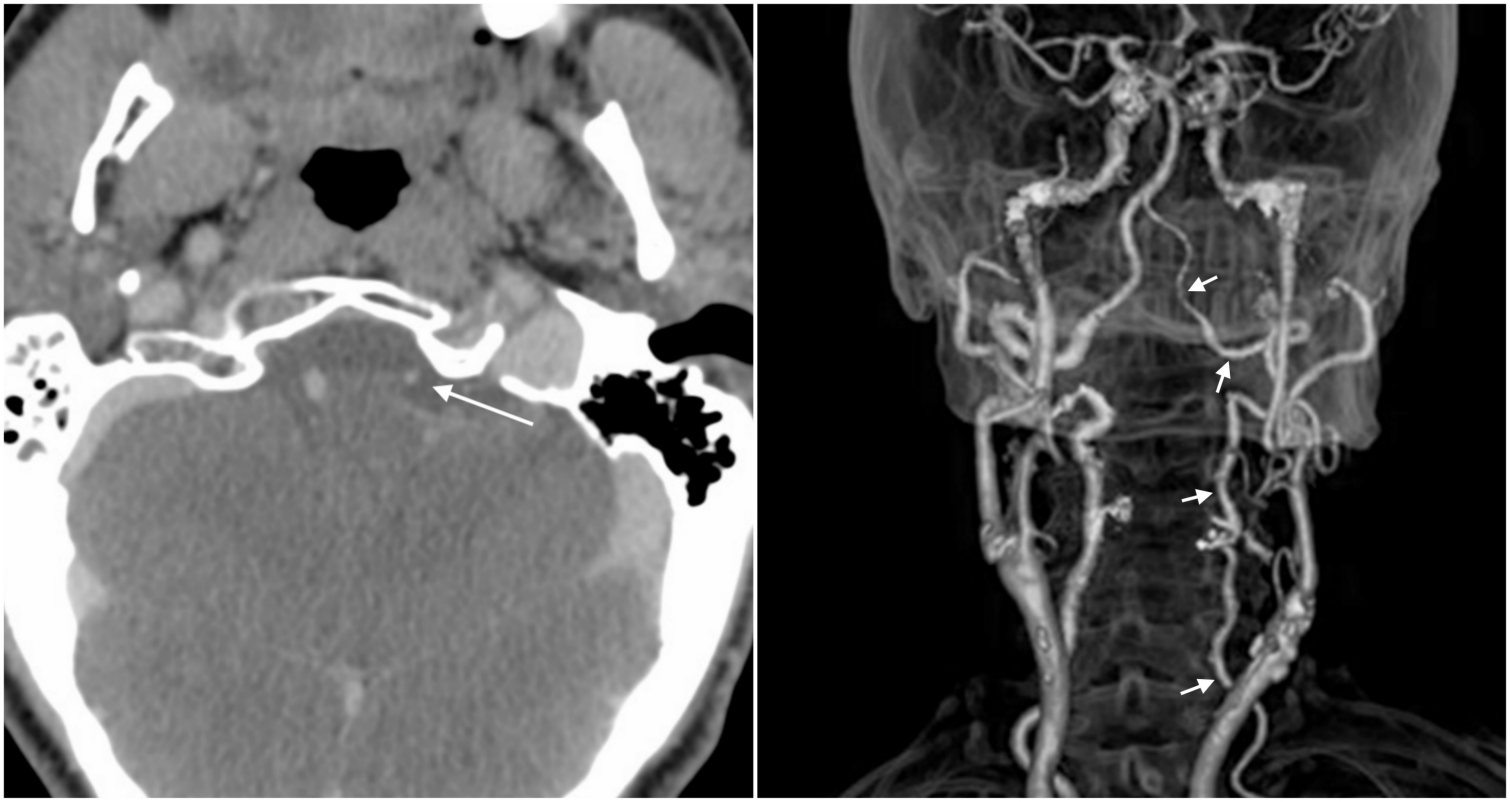
Computed tomography (CT) angiogram Left: Contrast CT scan showing the small diameter observed in the left vertebral artery (arrow). Right: CT 3D reconstruction displaying a hypoplastic left vertebral artery. This finding is observed from the origin of this artery (arrows).

An angio-CT scan was then performed to evaluate the status of the vertebral arteries. This test displayed asymmetry of vertebral arteries due to hypoplasia of the left artery (Figure 2).

The patient was discharged with medical treatment with clopidogrel and was sent to rehabilitation. One year later, he still experiences mild swallowing difficulty. In addition, he refers the presence of intermittent burning sensation on the right limbs which is being treated with gabapentin.

## Discussion

Lateral medullar syndrome (Wallenberg syndrome) usually causes a constellation of neurological signs either contralateral (trunk and extremities hypalgesia and thermoanesthesia) or ipsilateral (facial hypalgesia and thermoanesthesia; palatal, pharyngeal and vocal cord paralysis; Horner syndrome; cerebellar signs). It results from ischemia to the lateral medullary area most often caused by the intracranial vertebral artery or posterior inferior cerebellar artery occlusion.

It is reported that congenital asymmetry and hypoplasia of one of the vertebral arteries can be observed in up to 25% of healthy people [2]. In addition, it has been described that the patients with a hypoplastic vertebral artery have a higher risk of having an ipsilateral stroke in the territory of this artery [2]. In these cases, the propensity to ischemic stroke is higher because the blood flow through the hypoplastic artery is less than normal.

A percentage of patients with anaphylaxis can present with cardiovascular manifestations including tachycardia and hypotension. It is reported that approximately 20%-30% of patients with anaphylaxis may experience hypotension [6]. Some studies have reported patients who developed brain ischemia after bee stings [7-9] suggesting a mechanism of hypotension related to anaphylaxis as probable cause; however, it seems that the mechanism of stroke is very different. Bee venom itself contains histamine, thromboxane, leucotrienes, and other vasoactive and inflammatory mediators [7]. In addition, a neuropharmacological (sympathetic) mechanism of endothelial permeability involving the cerebral vasculature with a concurrent systemic thrombogenic has been suggested [8-9]. All this information suggests that brain ischemia secondary to bee stings has a different pathophysiology than that observed in our case.

In the current case, the hypotension caused by anaphylaxis very probably was an important factor that led to brain stem regional ischemia in the setting of preexistent ipsilateral VAH. The fact that the stroke occurred in a very specific territory supplied by a direct arterial branch of the hypoplastic vertebral artery strongly suggest this scenario.

VAH is a congenital anomaly which normally is asymptomatic. The presence of this condition is usually an incidental finding in patients with ischemic stroke when additional radiological tests are performed. Therefore, treating physicians can do little to change the presence of a preexistent vascular anatomical variant.

## Conclusions

This case shows that patients with hypotension secondary to anaphylaxis may have a higher risk of stroke, especially if a preexistent anatomical variation like VAH is present. Unfortunately, because VAH is a congenital anomaly, it is usually diagnosed after an ischemic stroke has occurred. Physicians treating these patients must be aware of this scenario.
